# Children with Imaginary Companions Focus on Mental Characteristics When Describing Their Real-Life Friends

**DOI:** 10.1002/icd.1869

**Published:** 2014-05-13

**Authors:** Paige E Davis, Elizabeth Meins, Charles Fernyhough

**Affiliations:** a Department of Psychology, Durham UniversityDurham, UK; b Department of Psychology, University of YorkYork, UK

**Keywords:** imaginary companions, friendship, mind-mindedness, theory of mind, peer relationships

## Abstract

Relations between having an imaginary companion (IC) and (i) descriptions of a real-life friend, (ii) theory of mind performance, and (iii) reported prosocial behaviour and behavioural difficulties were investigated in a sample of 5-year-olds (*N* = 159). Children who had an IC were more likely than their peers without an IC to describe their best friends with reference to their mental characteristics, but IC status was unrelated to children's theory of mind performance and reported prosocial behaviour and behavioural difficulties. These findings are discussed in the context of the proposal that there is a competence–performance gap in children's mentalizing abilities. © 2014 The Authors. *Infant and Child Development* published by John Wiley & Sons Ltd.

Young children's tendency to invent imaginary companions is well known. An imaginary companion (IC) may be entirely invisible or embodied in a toy or object (so-called personified objects), and as many as two-thirds of children report the existence of an IC at some point in childhood (Carlson & Taylor, 2005; Fernyhough, Bland, Meins, & Coltheart, 2007; Gleason, 2005; Gleason & Hohmann, 2006; Taylor, Carlson, Maring, Gerow, & Charley, 2004).

Taylor and Carlson (1997) were the first to propose that having an IC provides the child with greater opportunities to practise representing what others are potentially thinking. Subsequent research has supported this proposal, with studies showing how having an IC relates positively to children's tendency to represent people's differing perspectives. For example, Roby and Kidd (2008) investigated relations between IC status and children's communication by using an experimental paradigm to assess the child's ability both to convey and process verbal information (Camaioni, Ercolani, & Lloyd, 1995). The stimuli in each trial consisted of four pictures of the same entity varying on two dimensions (e.g. pictures of the same clown with a red or green collar, wearing a top hat or a bobble hat). In the speaker condition, the child was required to describe a particular picture in order to help the experimenter (who was behind a barrier) select it. In the listening condition, children had to select the picture when they were given either an ambiguous (e.g. wearing a top hat) or unambiguous (e.g. wearing a green collar and a top hat) description. Roby and Kidd found no IC-related differences in children's performance on the listening condition, but in the speaker condition, IC children were better able than their peers without ICs (NIC) to name a specific referent (e.g. the green collar) and to avoid describing redundant features. In a similar vein, Trionfi and Reese (2009) reported that IC-group children produced richer narrative accounts than their NIC peers both when telling a story and when narrating a personally experienced past event, despite the fact that the IC and NIC groups did not differ in their receptive or expressive linguistic abilities. Children with ICs are also better at understanding that other people are not the best judge of unobservable aspects of themselves, such as whether they are dreaming or feeling hungry (Davis, Meins, & Fernyhough, 2011).

Taken together, these findings show that children who create an IC have a better understanding of what information a listener or observer has and does not have access to, suggesting that they are better able to take others' perspectives into account. Moreover, ICs appear to fulfil similar social roles to children's real friends. Research suggests that ICs are not invented to compensate for children failing to make real-life friendships (e.g. Gleason, 2004; Gleason, Sebanc, & Hartup, 2000), and children appear to interact with ICs in similar ways to their interactions with real people (Gleason, 2002; Taylor, 1999). For example, Gleason and Hohmann (2006) found that there were no differences in children's reported interactions with ICs or friends with whom the friendship was reciprocated. However, children reported that they were more likely to want to play with or tell a secret to their IC than to a real friend with whom the friendship was not reciprocated. Recent research also suggests that interactions with ICs play a similar role to real-life social engagement in facilitating children's private speech development (Davis, Meins, & Fernyhough, 2013).

Despite these observed parallels between children's real and imaginary friendships, research has not yet addressed whether having an IC relates to how children think about and represent their real-world friends. Investigating this issue was the main aim of the study reported here. Specifically, we were interested in whether children's creation of an IC related to their tendency spontaneously to describe their real friends with reference to their mental characteristics rather than their physical appearance or behavioural traits. It was predicted that children with an IC would be more likely to invoke thoughts, desires, beliefs, and emotions in describing a real friend because of the greater salience of internal states afforded to them by engaging with their IC.

Individual differences in 7- to 9-year-olds' descriptions of a best friend were investigated by Meins, Fernyhough, Johnson, and Lidstone (2006) using an adaptation of the interview used to assess mothers' *mind-mindedness* (Meins, 1997) in describing their preschoolers (Meins, Fernyhough, Russell, & Clark-Carter, 1998). While all children mentioned friends' behavioural characteristics and over three-quarters included a physical description, only 54% of children used a mental attribute to describe their best friend. To explore potential reasons for individual differences in children's mental descriptions, Meins et al. (2006) investigated whether performance on the friend description task related to concurrent use of internal-state language when narrating a wordless picture book, and to children's theory of mind (ToM) abilities as assessed using Happé's (1994) strange stories task. Children's mental descriptions of their best friend were highly positively correlated with their use of internal-state language during the book-narration task, suggesting stability in children's tendency to invoke internal states either when describing a friend or when explaining and interpreting the events in a book. In contrast, internal-state language use on neither task related to children's ToM performance. Meins et al. argued that having a ToM does not necessarily mean that children will spontaneously use their knowledge of internal states when representing and interpreting other people and their behaviour. Apperly (2012) has also recently discussed this notion of a competence–performance gap in children's mentalizing abilities.

As well as exploring the link between IC status and children's descriptions of friends, the study reported here investigated whether there was a similar competence–performance gap between children's core ToM abilities and their tendency spontaneously to focus on internal states when describing a friend. The present study involved children who were younger (age 5) than the 7- to 9-year-olds in Meins et al.'s (2006) study; hence, we assessed children's ToM abilities by using a battery of standard ToM tests (Wellman & Liu, 2004) rather than the strange stories task. Including a ToM assessment also enabled us to attempt to replicate and extend a number of other findings in the extant literature.

First, we explored whether children's IC status related to their core understanding of false belief. Studies investigating relations between IC status and children's ToM performance present a mixed picture, with some studies suggesting that IC-group children outperform their NIC-group peers (Taylor & Carlson, 1997), while others find no relation between IC status and ToM (Davis et al., 2011; Fernyhough et al., 2007). However, there is no consistency in how ToM has been measured across these studies. Taylor and Carlson (1997) assessed ToM by using diverse tasks (e.g. appearance–reality, picture identification) that are not included in more recently standardized ToM batteries that focus on children's understanding of belief states (e.g. Wellman & Liu, 2004). Fernyhough et al. (2007) assessed ToM by using Flavell, Green, and Flavell's (1993) ‘stream of consciousness’ task, in which children have to demonstrate an understanding that people have ongoing thought processes even when they are not engaged in any obvious thought-provoking activity. Davis et al. (2011) used a standardized ToM battery, but the children in this study varied between 4 and 7 years, raising the suspicion that the ToM tasks were not appropriate for all of the children given that 7-year-olds are likely to be at ceiling on standard ToM tasks. Thus, no study has yet investigated whether performance on a standardized, age-appropriate ToM battery relates to children's IC status.

Finally, we investigated whether children's reported behaviour related to their IC status, ToM performance, and tendency to describe their friends with reference to their mental characteristics. Gleason (2004) argued that having an IC can potentially improve children's relationships with peers by enabling children to have more practice of peer interaction. It may be the case that engaging with an IC facilitates children's real-life prosocial behaviour, which in turn may relate to children's ToM and tendency to focus on their real friends' mental characteristics. No study has investigated how children's IC status relates to their prosocial behaviour with peers. Indeed, as Caputi, Lecce, Pagnin, and Banerjee (2012) noted, studies investigating social relationships and children's understanding of other minds are surprisingly rare. The study reported here was thus the first to explore how having an IC related to children's behaviour with peers, and focused both on reported prosocial behaviour and behavioural difficulties.

In summary, the present study investigated how children's IC status related to their descriptions of a best friend, performance on a battery of ToM tasks, and reported prosocial behaviour and behavioural difficulties. It was hypothesized that having an IC would be positively associated with mental descriptions of friends. We also investigated how IC status related to children's ToM performance and reported behaviour, although no directional hypotheses were made regarding these associations.

## METHOD

### Participants

Participants were a socially diverse sample of 159 children (81 girls) who had been participating with their mothers in a longitudinal study that began in the child's first year of life (Meins, Fernyhough, Arnott, Leekam, & Turner, 2011; Meins, Fernyhough, Arnott, Leekam & de Rosnay, 2013; Meins et al., 2012; Meins, Muñoz Centifanti, Fernyhough & Fishburn, 2013). Family socio-economic status (SES) was assessed using the Hollingshead Index (Hollingshead, 1975), with scores ranging from 14 to 66 (*M* = 35.78, *SD* = 14.03). Children were aged between 59 and 64 months (*M* = 61.3 months, *SD* = 1.08 months). Parents gave full informed consent for their children to be tested and recorded in their schools.

### Materials and Methods

Children were tested individually by a female experimenter in a quiet area in their school. Tasks were administered in the order described below.

#### Theory of mind

A battery of ToM tasks was administered to each child. The battery consisted of six tasks used extensively in the field, based on Wellman and Liu (2004): (i) Diverse Beliefs task to assess children's understanding of non-egocentric beliefs, (ii) Knowledge Access task to assess children's understanding of the relation between seeing and knowing, (iii) Contents False Belief–Other task to assess children's ability to override their own knowledge of the (unexpected) contents of a container in predicting a naïve child's belief about its contents, (iv) Contents False Belief–Self task to assess children's ability to override their current knowledge of the (unexpected) contents of a container and recognize their own initial false belief about its contents, (v) Explicit False Belief task to assess children's ability to predict the protagonist's search behaviour on the basis of what they are told about where he or she thinks the object is rather than where the object really is, and (vi) Unexpected Transfer task to assess children's ability to predict the protagonist's behaviour on the basis of his or her false belief.

The order in which the stories were presented was randomized and counterbalanced, and the gender of the story protagonists matched that of the child. Memory and reality control questions were used, and all control and test questions had to be passed for the child to be credited with passing each task. For each task passed, the child received 1 point, resulting in total possible scores ranging from 0 to 6 points. The internal reliability of the ToM battery was satisfactory, Cronbach's *α* = .68, and in line with those of studies employing similar ToM measures (e.g. Astington & Jenkins, 1999; Meins et al., 2002). Total scores out of 6 were used in the analyses.

#### Descriptions of best friend

The experimenter first asked the child if he or she had a best friend. No child reported that he or she did not have a best friend. After the child named their best friend, he or she was asked, ‘Can you tell me about [friend's name]?’ When the child finished the description, the experimenter asked, ‘Is there anything else you would like to tell me about [friend's name]?’ Children were audio-taped while describing their best friend, and the descriptions were later transcribed verbatim. The descriptions were divided into discrete descriptions that could be single words, phrases, or sentences.

The protocol described by Meins et al. (2006) was used to code children's mental descriptions of their friends. The transcripts were coded by a researcher who was blind to all other data. Each description was placed into one of Meins et al.'s exclusive and exhaustive categories: (i) Mental: references to the friend's desires, emotions, cognitions, intellect, and the friend's responses to the child's own internal states (e.g. ‘He's kind when I hurt myself’); (ii) Behavioural: references to activities or interactions that could be interpreted on a purely behavioural level (e.g. ‘She rides a bicycle’, ‘He plays with me’), (iii) Physical: references to physical characteristics, age, or position in the family (e.g. ‘He has light brown hair’, ‘She's got a sister called Kate’), (iv) General: any comment that did not fit into the categories above (e.g. ‘He's got a big garage’). Describing the friend as ‘nice’ was included in the general category if no other information was provided to qualify how to characterize being ‘nice’.

A randomly selected 30% of the transcripts was coded by a second researcher who was blind to all other data and to the hypotheses of the study; inter-rater reliability was *κ* = .75. Children received a score for the total number of mental descriptions and for mental descriptions as a percentage of the total number of friend descriptions. Percentage scores were used in the analyses to control for individual differences in verbosity. (Note, however, that the exact same pattern of findings emerged if frequency scores were used.)

#### Imaginary companion interview

The interview developed by Taylor and Carlson (1997) was used to assess children's IC status. Children were asked by the researcher about their pretend friends. The researcher began,

‘Now I am going to ask you some questions about friends. Some friends are real, like the kids who live on your street, the ones you play with. And some friends are pretend friends. Pretend friends are ones that are make-believe that you pretend are real. Do you understand?’

When the child indicated understanding, the researcher went on to ask if the child had a pretend friend. If the child indicated the existence of an IC by responding affirmatively, they were asked: (i) its name; (ii) whether people other than the child could see the IC; (iii) whether the child could see the IC; (iv) its gender, age, and physical appearance; (v) what the child liked and disliked about the friend; and (vi) where the friend lived and slept.

The children's mothers separately completed a parental questionnaire on whether or not the child had an IC. In addition to the questions on the child's IC interview, mothers were asked to indicate how long the child had had the IC and whether it was entirely imaginary or personified in a toy or object. These data from the mothers enabled us to establish whether the child's IC was corroborated by a parent. Children were credited with having an IC either if the IC was corroborated by the mother or if the child provided a convincing, fleshed-out description of the IC. All of the children who reported ICs that were not corroborated by the mother explained in the interview that the IC was known only to them, often saying that its existence was secret. Two independent coders rated the veracity of the non-corroborated ICs, achieving perfect agreement.

#### Receptive language ability

Children's receptive verbal ability was assessed at age 51 months using the British Picture Vocabulary Scale (BPVS; Dunn, Dunn, Whetton, & Burley, 1997) to control for any effects of verbal ability on ToM performance or children's friend descriptions. Verbal ability data were available for 134 children. The mean standardized score was *M* = 102.65, *SD* = 12.86, range 43–132.

#### Children's reported behaviour

Children's behavioural difficulties were assessed using the Strengths and Difficulties Questionnaire (SDQ; Goodman, 1997), which was completed by the child's teacher and the child's mother. The SDQ includes 25 items that are each rated on a 3-point scale (‘not true’, ‘somewhat true’, and ‘certainly true’), and is suitable for children aged between 3 and 16 years. Children's behaviour is rated on five separate scales: (a) emotional symptoms (e.g. worried, nervous, fearful, or unhappy behaviour), (b) conduct problems (e.g. temper tantrums, fighting, bullying, lying), (c) hyperactivity (e.g. restlessness, concentration difficulties, impulsivity), (d) peer problems (e.g. solitary behaviour, being picked on or bullied), and (e) prosocial (e.g. considerate to others' feelings, shares readily). Scores on subscales (b) and (c) are totaled to give an externalizing behaviour score (range = 0 to 20), and scores on subscales (a) and (d) are totaled to give an internalizing behaviour score (range = 0 to 20), with potential prosocial scores ranging between 0 and 10. Following Kamphaus and Frick (2002), the SDQ data from mothers and teachers were used to create resolved scores, such that the child was credited with the higher score for a particular SDQ item if it was endorsed by either respondent.

## RESULTS

### Descriptive Statistics and Preliminary Analyses

Sixty-eight children (43%) reported having an IC, 34 of which were corroborated by their mothers. Of the 68 ICs, 47 were invisible; of the 34 corroborated ICs, 23 were invisible. The patterns of effect were the same when analyses were run using the maternally corroborated and total IC groups, and so only analyses involving all 68 children in the IC group are reported below.

Mothers reported that the IC had existed for between 2 and 48 months, with the average duration of existence being 25.19 months (*SD* = 12.65 months). Thus, all ICs that were corroborated by mothers were long-standing. Children in the invisible IC and personified object IC groups did not differ on any of the variables (*t*s < .90, *d*s < .24), and so these two categories were collapsed in the analyses.

Seventy children (44%) used at least one mental characteristic to describe their best friend. Percentage scores for mental descriptions were non-normally distributed. Spearman's ρ was thus used for the correlational analyses, but analysis of covariance (ANCOVA) was used since the *F*-test is robust against violations of the assumption of normality as long as there are at least 20 degrees of freedom for error (Tabachnick & Fidell, 2007).

Children's IC status was marginally related to SES, with children in the IC group coming from higher SES backgrounds (*M* = 38.61, *SD* = 12.61) than children in the NIC group (*M* = 34.02, *SD* = 14.80), *t*(157) = 1.95, *p* = .053, *d* = .33.

Child gender was unrelated to IC status, *χ*^2^(1) = 0.01, *p* = .911, to reported internalizing behaviours, *t*(157) = 0.97, *p* = .336, *d* = .17, and to BPVS scores, *t*(132) = 0.50, *p* = .619, *d* = .09. Girls (*M* = 8.74, *SD* = 1.54) were reported to be more prosocial than were boys (*M* = 7.74, *SD* = 2.05), *t*(157) = 3.00, *p* = .003, *d* = .55, and girls (*M* = 4.35, *SD* = 1.56) achieved marginally higher ToM scores than did boys (*M* = 3.83, *SD* = 1.72), *t*(157) = 1.86, *p* = .066, *d* = .32. Boys (*M* = 25.69, *SD* = 31.73) achieved marginally higher scores for percentage of mental descriptions of friends than did girls (*M* = 17.53, *SD* = 24.13), *t*(157) = 1.72, *p* = .088, *d* = .29. Boys (*M* = 3.95, *SD* = 3.11) were also reported to have marginally higher levels of externalizing behaviours compared with girls (*M* = 2.77, *SD* = 3.44), *t*(157) = 1.93, *p* = .056, *d* = .36.

Children's ToM scores were positively correlated with BPVS scores, *r*(130) = .40, *p* < .001, and reported prosocial behaviour, *r*(156) = .18, *p* = .045, and were negatively correlated with reported externalizing behaviours, *r*(156) = −.25, *p* = .002. ToM scores were unrelated to reported internalizing behaviours, *r*(156) = .12, *p* = .143.

### IC Status and Mental Descriptions of Best Friends

Table1 shows the mean scores for percentage of mental descriptions as a function of IC status. The relation between children's IC status and their tendency to describe a best friend with reference to mental characteristics was investigated in a 2 (IC, NIC) × 2 (male, female) ANCOVA with children's receptive verbal ability and SES entered as covariates.

**Table 1:** Mean (standard deviation) friend description, theory of mind, receptive verbal ability, and child behaviour scores as a function of imaginary companion status

	No imaginary companion	Imaginary companion
Percentage of mental descriptions	12.61 (20.37)	29.63 (32.57)
Total number of descriptions	4.54 (2.28)	4.94 (2.60)
Theory of mind	3.96 (1.67)	4.23 (1.64)
British Picture Vocabulary Scale	103.55 (12.19)	103.07 (13.74)
SDQ externalizing behaviours	6.83 (4.17)	6.60 (3.46)
SDQ internalizing behaviours	3.88 (2.80)	4.14 (3.50)
SDQ prosocial behaviour	7.87 (2.03)	8.34 (1.80)

SDQ, Strengths and Difficulties Questionnaire.

There was a main effect of IC status, *F*(1, 127) = 20.92, *p* = .001, *η*^2^ = .141, a marginally significant main effect of gender, *F*(1, 127) = 2.92, *p* = .090, *η*^2^ = .020, and no IC status × child gender interaction, *F*(1, 127) = 0.02, *p* = .897, *η*^2^ = .001. Controlling for receptive verbal ability, SES, and gender, children in the IC group were more likely than their NIC-group peers to describe a best friend with reference to mental characteristics.

### IC Status and Theory of Mind

Table1 shows the mean ToM scores as a function of IC status. The relation between children's IC status and their ToM performance was investigated using ANCOVA as above. There was a main effect of child gender, *F*(1, 127) = 4.23, *p* = .040, *η*^2^ = .029, no main effect of IC status, *F*(1, 127) = 0.05, *p* = .823, *η*^2^ = .003, and no IC status × child gender interaction, *F*(1, 127) = 0.16, *p* = .692, *η*^2^ = .001. Controlling for receptive verbal ability, SES, and IC status, girls performed better on the ToM task than did boys.

### IC Status and Reported Child Behaviour

Table1 shows the mean SDQ externalizing, internalizing, and prosocial behaviour scores as a function of IC status. The relation between children's IC status and their reported behaviour was investigated using ANCOVA as above. For SDQ externalizing behaviours, there was a main effect of child gender, *F*(1, 126) = 8.20, *p* = .005, *η*^2^ = .057, no main effect of IC status, *F*(1, 126) = 0.11, *p* = .736, *η*^2^ = .001, and no IC status × child gender interaction, *F*(1, 126) = 1.77, *p* = .186, *η*^2^ = .012. Controlling for receptive verbal ability, SES, and IC status, boys were reported to have more externalizing behaviours than were girls.

For SDQ internalizing behaviours, there was no main effect of IC status, *F*(1, 126) = 0.76, *p* = .386, *η*^2^ = .006, no main effect of child gender, *F*(1, 126) = 0.14, *p* = .708, *η*^2^ = .001, and no IC status × child gender interaction, *F*(1, 126) = 1.48, *p* = .227, *η*^2^ = .012.

With respect to SDQ prosocial behaviours, there was a main effect of child gender, *F*(1, 126) = 9.97, *p* = .002, *η*^2^ = .086, no main effect of IC status, *F*(1, 126) = 1.35, *p* = .248, *η*^2^ = .012, and no IC status × child gender interaction, *F*(1, 126) = 0.94, *p* = .336, *η*^2^ = .008. Controlling for receptive verbal ability, SES, and IC status, girls were reported to behave more prosocially than were boys.

### Correlates of Mental Descriptions of Best Friends

Children's tendency to describe a best friend with reference to their mental characteristics was unrelated to concurrent ToM performance and reported SDQ externalizing behaviours, internalizing behaviours, and prosocial behaviours (*ρ*s < .09).

## DISCUSSION

The main aim of the study reported here was to investigate how having an IC related to children's tendency spontaneously to focus on mental characteristics when given an open-ended invitation to describe a best friend. The results showed that children with ICs were more likely than their NIC peers to describe their best friend with reference to mental characteristics. Our second aim was to investigate how children's IC status related to their performance on a battery of tasks assessing core ToM abilities. No association was found between ToM performance and IC status, replicating the null findings of Fernyhough et al. (2007) and Davis et al. (2011). In contrast, the lack of association between IC status and ToM is at odds with Taylor and Carlson's (1997) finding that children with ICs showed superior ToM abilities compared with their NIC counterparts. However, as mentioned in the Introduction, the ToM assessment in Taylor and Carlson's study differed from that used in the present study. Two of the three tasks used to assess ToM in this earlier study involved understanding different perspectives (e.g. the difference between appearance and reality) rather than belief states. Thus, it may be the case that having an IC was positively related specifically to children's understanding of perspective-taking, and this may explain the discrepancy in findings. Taylor and Carlson did not report how IC status related to performance on the individual ToM tasks, but it would be interesting to explore in future research whether children with ICs have a specific advantage in understanding diverse perspectives rather than diverse beliefs.

We also replicated Meins et al.'s (2006) null finding on the relation between ToM performance and children's tendency to describe their friends with reference to mental characteristics. The present study assessed core ToM abilities in a sample of 5-year-olds, whereas Meins et al. assessed understanding of more sophisticated aspects of mind (e.g. faux pas, white lies) in 7- to 9-year-olds. Given that both studies found no association between the ToM measure and children's mental descriptions of friends, it would appear that the null findings cannot be explained in terms of children's age or method of ToM assessment. Moreover, replicating well-established relations (e.g. Astington & Jenkins, 1999; Hughes & Ensor, 2007), we found that children's ToM scores were positively correlated with their receptive verbal ability and negatively correlated with reported externalizing behaviours. It would thus appear unlikely that our assessment of children's ToM abilities was invalid or unreliable.

The fact that mental descriptions of friends did not relate to ToM performance suggests that the basic ability to understand the relation between beliefs and behaviour is not equivalent to the tendency in everyday life to represent people in mentalistic terms—the latter quality is what Meins (1997) termed mind-mindedness. Our findings are thus consistent with the view that there is a competence–performance gap in mentalizing abilities (Apperly, 2012; Meins et al., 2006). The observed pattern of findings is also in line with the results of a series of studies reported by Meins, Fernyhough, and Harris-Waller (2014), which suggested that mind-mindedness is a quality of personal relationships. Meins et al. (2014) found robust positive correlations between adults' mental descriptions of different people with whom they had close relationships (e.g. child, romantic partner, close friend). However, the tendency to describe a significant other with reference to mental characteristics was unrelated to individuals' tendency to focus on such characteristics when describing famous people or works of art. Furthermore, individuals were more likely to include mental characteristics when describing someone with whom they had a close relationship, leading Meins et al. to argue that mind-mindedness is not a trait-like quality, but a facet of personal relationships. We should thus not be surprised by the observed lack of association between children's mental descriptions of a best friend and their general mentalizing abilities.

A further aim of the present study was to explore how children's reported prosocial behaviour and behavioural difficulties related to both their IC status and their tendency to focus on their friends' mental characteristics. No associations were found between children's reported behaviour and either IC status or friend descriptions. In addition, although the positive correlation between reported prosocial behaviour and children's ToM was significant, the effect size for this relation was small. Taken together, these findings suggest that children's grasp of internal states is not necessarily translated into a greater awareness of people's motives and intentions in everyday interactions. Indeed, given the research showing high-level ToM understanding in ringleader bullies (Sutton, Smith, & Swettenham, 1999), a good grasp of the link between beliefs and behaviours may be used in more Machiavellian ways to manipulate and dominate others.

The findings reported here suggest several avenues for future research. The observed positive association between having an IC and tending to focus on mental characteristics in describing a best friend is consistent with the notion that having an IC entails that the child becomes practiced in focusing on cognitions and emotions (Taylor & Carlson, 1997). Further, our findings show that this focus on internal states appears to generalize to how children represent their real-life friends. However, given that IC status and children's friend descriptions were assessed concurrently, the opposite direction of cause and effect should also be considered: it may be that children's tendency to emphasize their friends' mental and emotional characteristics will make them more likely to invent an IC. Although the present study's concurrent data cannot provide a definitive answer to the question of cause and effect, the fact that ICs were reported by the mothers to have existed for a large proportion of the children's lives (over 25 months on average) suggests that inventing an IC is unlikely to be the result of children's mind-mindedness in relation to their friends. Indeed, because these children were in the first year of school and describing friends whom they may have known for a relatively short period of time, it is likely that the ICs will have predated the establishment of these friendships. Future research should explore longitudinal relations between the creation of an IC, the establishment of real-world friendships, and children's mental descriptions of friends in order to establish the true direction of cause and effect.

Exploring individual differences in children's ICs is also worthy of future research attention. ICs differ in the extent to which they are perceived to lead lives independently of the children who create them; some are replicas of their child creators, whereas others are very different and reportedly behave in ways that their creator dislikes, disapproves of, or is surprised by (see Taylor & Carlson, 1997; Taylor, Hulette, & Dishion, 2010). It may be that children who have ICs who they deem to behave badly will be less likely to recognize prosocial acts in their peers and behave in prosocial ways themselves. In investigating this issue, it would be worthwhile to assess how children spontaneously describe their ICs as well as their real-life friends to investigate concordance in the types of characteristics mentioned. Research on ICs asks children various questions about the IC's appearance and behaviour, but to our knowledge, no study has yet assessed the characteristics that children spontaneously focus on when given an open-ended invitation to describe the IC. Studies exploring heterogeneity within IC-group children could thus enrich our understanding of how imagination relates to children's real-world relationships with peers.

Finally, the study reported here relied solely on adult report to index children's behavioural difficulties and prosocial behaviour. The null findings on relations between children's behaviour and both their IC status and tendency to describe friends with reference to mental characteristics should thus be treated with a degree of caution. Future research should explore these relations further by obtaining observational measures of children's behaviour or using experimental tasks to assess children's tendency to behave prosocially. These more in-depth assessments of peer interaction will provide a clearer picture of how children's engagement with an IC and their tendency to focus on their real friends' mental characteristics relate to their actual behaviour with peers.
